# Academy of Stomatology

**Published:** 1897-03

**Authors:** 

**Affiliations:** Academy of Stomatolgy


### ACADEMY OF STOMATOLOGY.

A regular meeting of the society was held at its rooms, December 22, the President, Dr. James Truman, in the chair.

After the routine business, the reports of committees were called for.

Clinic Committee, Dr. Register, chairman, reports two clinics of the afternoon,—one, by Dr. F. D. Gardiner, upon the use of heavy rolled foils and the comparative merits of the electric, Register's, and Bonwill's mechanical mallets as welding instruments; the other, by Dr. H. E. Roberts, upon a novel and inexpensive electrical outfit.

Dr. Gardiner stated that the case upon which he operated and the lateness of the hour combined to make any actual demonstration of the subject incomplete and unsatisfactory. The preference of one form of mallet over another he believes to be more a question of individual familiarity with the instrument itself than of intrinsic superiority of any of the mallets. Each man chooses that which will best serve his taste. To one this will mean the electric mallet, to another, one of the engine mallets.

In manipulating heavy rolled foil it is to be remembered that we are dealing with gold plate, not hammered foil, and our work should be done with a recognition of the physical properties of the material. Like all gold plate, it should be annealed to redness, as any heat below this does not develop the full degree of cohesiveness. Instead of making the gold harsh and hard, as with foil, the heat actually softens it, makes it more plastic. The numbers of foil used in the clinic to-day were 60 and 120. To use these numbers requires absolute precision in manipulation, although perfect welding is accomplished by means of very light blows; each succeeding piece must be added to its predecessor without folding or wrinkling. Rolled foil is best adapted for use in spaces which have a narrow approach. When cut in narrow strips it may be carried to the depths of the cavity, and when the proper care is exercised be perfectly adapted. Another advantage possessed by it is its perfect welding under slanting blows.

*Dr. H. E. Roberts.*—The outfit I exhibited seemed to me to meet the requirements of the dentist better than any I had seen. The motor is small and comparatively noiseless, and is attached directly to the flexible arm or shaft. Other attachments for the cord engine can easily be made. It is most carefully and thoroughly made, and of exceptionally high efficiency, consuming only about one-thirtieth of one-horse power of electrical energy and giving ample power. One distinctive feature is that the armature runs on a hollow shaft, through which passes a shaft having a light iron disk, which is attracted to the armature, making a magnetic clutch when the current is turned on, but which is loose and runs free when the current is off. The other end of the shaft is attached to the flexible cable. When any stress is upon the bur and the current turned off to stop the motor, the armature will run free and the bur come to a stand-still, with no power to drive it. The motor, as shown, is wound to go with a new storage battery, which is novel and simple in construction, and forms a very important adjunct in the efficiency of the outfit. It is made of a series of lead plates (fourteen in number), laid one upon the other, with a separating medium to hold a small amount of acid, and it makes practically a dry cell. Each plate represents two volts, one side being negative and the other positive, and as there are fourteen plates, we have twenty-eight volts in one box about twelve by twelve by twenty inches, giving twenty-five ampère hours. They are made in other sizes. The active material cannot fall from the plates. It is claimed that the battery can be short-circuited without injury, and in theory the longer it is used the more efficient it should become, until the whole substance of the plate is reduced to active material, taking a long time. The battery, being of twenty-eight volts, is admirably adapted for cataphoresis, and the manufacturers have gotten out the neatest (and apparently very efficient) volt-selector and regulator that I have seen. The plant was put in for me by Messrs. Louis Costa, Jr., & Co., 1227 Callowhill Street, the manufacturers, who also make other surgical and dental electrical appliances.

Upon the motion of Dr. Louis Jack, the date of clinics was changed to 2 P.M. on Saturday preceding the regular meetings.

*The President.*—We will now listen to the paper by Dr. S. H. Guilford.

(For Dr. Guilford's paper, see page 85.)

DISCUSSION.

*The President.*—You have listened to this clear and instructive paper of Dr. Guilford's; the subject is now open for discussion.

*Dr. Burchard.*—I do not think sufficient attention is directed by the dental fraternity to the grave question of the influence of dental pain upon mastication and nutrition in children. While the temporary teeth are developing, erupting, and in position, the alimentary canal and all of its glandular appendages are also undergoing developmental transformation, and influences, such as imperfect mastication, which would have an injurious effect upon the gastric and intestinal digestion in mature organisms, would certainly be fraught with greater danger when influencing organs which are in a developmental state. This in itself is serious enough matter, aside from local considerations, to warrant the close care of the temporary teeth.

The indications for the treatment of children's teeth are quite as clearly outlined as those for the permanent teeth; but there are unfortunate limitations due to the anatomical structure of the mouth and its organs, and the age of the child, which prevent us doing what we should. We cannot do exactly what is indicated, but do only what we can.

There is one consideration which is of importance, and that is the anatomical organization of the tissues of the temporary teeth as compared with the permanent teeth. Tooth development, or the elaboration of the hard tissues of the temporary teeth, beginning at approximately the fifth or sixth month of intra-uterine life, is completed in the third, fourth, and sometimes fifth year. The development of the permanent teeth, from the formation of the enamel organs of the first permanent molar to the eruption of the second permanent molar, extends from the fourth month of intra-uterine life to the thirteenth or fourteenth year; the formation of one a matter of months where the other is a matter of years,—that is, there is a higher or more advanced organization of the tissues of the permanent teeth. Dr. Black's investigations have shown that the chemical analysis of teeth, as regards the amount of calcium salts contained in them, is not sufficient to explain the greater physical or, more particularly, the chemical differences which exist between teeth. While no one can question the accuracy of Dr. Black's analyses, every clinician recognizes the greater resistance offered by some teeth than others in cutting them. Studying Dr. Black's work, in connection with that of Dr. J. L. Williams, in the *Cosmos* of this year, I have become impressed that the differences in teeth are due to the anatomical organization of their several tissues,—that is to say, given a certain intimacy and degree of combination of calcium salts with albuminous material, to form the most resistant varieties of dentine and enamel, less resistant specimens would represent a lessened intimacy of chemical combination; they are insufficiently built or elaborated; the higher types have a higher anatomical organization.

I believe that Dr. Williams will at some time furnish us with a proof of this hypothesis.

I do not recognize the basis of objection to the use of zinc phosphate in temporary teeth. It can affect the teeth in one or more of three ways,—by the action of free acid sodium phosphate which it may contain. This is readily prevented by varnishing a cavity before inserting the cement; it might exercise pressure; the same objection obtains with any filling material. Its moderate conductivity might be a source of disturbance to the pulp, but I have never heard it complained of. Kept free from contact with lactic acid, the dental solvent, it disintegrates but slowly, therefore lasts well and does excellent service upon the masticating surfaces.

of teeth. In approximal spaces it, of course, disintegrates, but still is sometimes useful there.

The uses of gutta-percha, amalgam, and, under favorable conditions, tin in the temporary teeth are indorsed almost universally.

We frequently hear the expression, "The large open canals of temporary teeth." Any one may demonstrate conclusively that there is an error here. Make some sections of the roots of temporary molars, and while you will find the pulp-chamber relatively large, as a rule, the canals are not large, and may be thin and tortuous. I mean, of course, in fully-formed roots, before resorption begins.

When we are confronted with abscess upon temporary teeth, not infrequently we have marked examples of the swift reactions of the infantile system. I have noted many cases of septic intoxication in connection with abscess upon the temporary teeth, and in some instances marked septic poisoning, rigors, a fever with temperature 104°, some delirium, and disturbances of the alimentary tract. These cases should have early evacuation and thorough syringing with pyrozone, three per cent., followed by an injection of a ten-per cent. solution of meditrina, and the symptoms usually vanish. After sterilization and the subsidence of the inflammatory symptoms, the roots of these teeth should be filled with balsamo del deserto if you can manipulate it; nearly all of my attempts to use it, following the directions given, have resulted in dismal failure, and recourse was had to salol or paraffine. Aristol is a good medicinal dressing for these cases.

In cavities which cannot be given a retentive form, the advance of caries may be prevented by rubbing the surface of the dentine with fused nitrate of silver. A portion of the salt is placed upon a platinum wire and held over a flame until fused into a button as described by Dr. Craven, this is to be rubbed freely over the dentine. Of course it discolors, but that is a minor evil in the temporary teeth.

*Dr. Register.*—I also believe in the importance of the relation between sound temporary teeth and the proper development of the digestive apparatus. Carried to its logical end we should find this a very serious matter.

To avoid the decay of the mesio-approximal surface of the first permanent molars, near the point of contact with the second temporary molars, it has been my practice to dress away the distal wall of the latter by means of a disk, leaving a shoulder at the neck to preserve a space between the crowns. I use iodine freely upon the crowns of temporary teeth as a cleansing agent; to remove the green deposits I give the prescription,—

> ℞ Tr. iodi. co., ʒi;

> Glycerini, ℥ii;

> Ol. menth. pip., q. s. for flavoring. M.

This is to be painted upon the surfaces of the teeth by nurse or patient, and it does good service.

*Dr. W. H. Trueman.*—This essay would make very instructive and useful popular reading. Parents should be impressed with the importance of the facts given. You know we do not have dealings with ideal parents, although the latter always have in their own opinions ideal children, and this is a hard condition to contend with. I recall cases which are of interest in connection with the subject of the essay.

The first, that of a child whose teeth very shortly after eruption crumbled and wore away until the entire crowns were gone, and this was due more to faulty structure than to any diseased processes. The permanent teeth when erupted were good both in texture and form.

The second case I had where a marked mental impress resulted from a conspicuous facial deformity. A lad of about fifteen was brought to me. The day before, his elocution teacher had discovered a marked malocclusion that, strange to say, no one at home had noted, and this was his first visit to a dentist. He had been a delicate child, having been unable to walk, owing to imperfect development of the bony tissues, until about his seventh year. At this time his health was fairly good, and physically he seemed well developed. His mother informed me that he was of a very peculiar disposition, which they attributed to injury at birth, and to that also they attributed in a measure his delicate health during childhood. While he made fair progress in his studies at school he would not mingle with other children, took no interest in their sports, and at home showed a strong aversion to company; indeed, took but little interest in anything. On examination I found that in occlusion the second molars alone touched, and that the front teeth, when these molars were in close contact, did not meet by nearly three-quarters of an inch. He could not approximate the lips, and this gave him a marked idiotic expression. He seemed to be constantly making an effort to bring the lips together, thus making the deformity more conspicuous. I found him quite apathetic. He took but little interest in the examination, or the suggestions made, and impressed me as being the idiot that he looked. The second molar teeth were extracted, and the first molars ground as short as prudence permitted. As an immediate result, the occlusion was so far improved that the bicuspid teeth became useful in mastication, the incisors approximated to within about a quarter of an inch, and the lips could be comfortably closed. The change in his expression was very great,—not more so, however, than the change in the boy: it was a transformation. It is a marvel to me that the cause of so marked a deformity should have so long escaped the notice of an anxious, watchful mother and that of the family physician, a near relative and a man of ability and experience, under whose care he frequently was.

How the deformity developed I could obtain no information; its existence was to them a surprise. I have no doubt that it was hereditary. I had a few years before made for his father a full denture, and had a great deal of trouble owing to the very marked disparity in the length of the posterior and anterior teeth. At the time the boy came into my care the father was suffering from paralysis, and, other than that he had been edentulous for many years, I was unable to obtain anything regarding his dental history. In this case the anterior teeth continued to approach, so that after the lapse of a year or two, without farther attention, they nearly met, and now the occlusion is fairly good. The mental peculiarities disappeared within a short time after he found that he could mingle with others without being stared at, and he became like other boys.

The third case is curious as exhibiting the effect of morbid sensitivity as to a physical blemish upon the mind. A child who was a thumb-sucker from infancy developed a condition of anterior protrusion of the upper teeth which amounted to a marked and very disfiguring deformity. When she attained an age when a physical blemish would be most noticed she developed a morbid sensitiveness as to her deformity, which so reacted upon her mind as to render her almost imbecile.

The work of Dr. Black, cited by Dr. Burchard, valuable as it is from a scientific point of view, has as yet but little clinical application, although it certainly will have in the future. All of us have noticed the difference of the resistive power of teeth to cutting instruments; some of them cut like soft wood, others almost or quite give sparks in cutting.

*Dr. J. A. Woodward.*—This subject is one of special interest to me. My practice in reference to the second temporary molar has been to cut it away only when a strong shoulder can be left to retain the space and protect the gum between it and the first permanent molar.

When decay has so far progressed that this is not possible, I restore the contour of the temporary molar in amalgam. Should the permanent molar have a cavity in the mesial surface, that is filled with gutta-percha until the temporary molar is lost, when it can be easily contoured in gold previous to the eruption of the second bicuspid.

After treating abscesses and sterilizing, the roots of temporary teeth are filled with oxychloride. When their permanent successors appear the crowns of these teeth have very frequently been found to be rootless, resorption at times having uncovered the filling material in the root canals and floor of space occupied by the pulp.

*Dr. Gaskell.*—I have noted the perfect resorption of the root of a temporary tooth which had been violently displaced by a blow; the tooth was replaced, and lost at the age of eight years; the resorption of the root was complete.

Dr. Roberts reported a similar case.

*Dr. Darby.*—One of our great difficulties in dealing with the teeth of children is the devitalization of the pulps when indicated. I have used, and with much success, for this purpose a paste of powdered cantharides and carbolic acid; say about one-twentieth grain of the powder with enough carbolic acid or creosote to make a paste. I know that the use of arsenic for this purpose is justly viewed with much suspicion, but my opinion is that it is largely a question of how much arsenic is used. I use arsenic for this purpose in very minute quantities and have had no ill results. The canals of children's teeth should of course be cleansed thoroughly and sterilized. I question the use of cotton dressings in these cases, for should the foramen be large, owing to a partial resorption of roots, soft tissues might be impinged upon, and the cotton becomes a source of irritation or worse. I think the safer practice is to use fluid in the canals and oxychloride in the pulp-chamber. An old practice was to fill the crown cavity and drill a vent-hole through the root. This has been abandoned for good and sufficient reasons.

*Dr. Burchard.*—Touching this question of the resorption of the roots of temporary teeth, it is brought about by multinucleated cells, which are named from their function odontoclasts,—that is, the removal of the roots is a species of phagocytosis. The process begins usually upon some lateral aspect of the root near the apex, not at the apex. Now, if the tissues of the pericementum be healthy, the process goes on uninterruptedly; if they are the seat of disease, this cellular function is not performed. The most prominent source of disturbance is debility of these cells from poisoning by the action of the waste-products of pathogenic organisms, notably the pyogenic organisms. If this poisoning is prevented by absolute sterilization, the resorption occurs even in the absence of the pulp, as has been shown here this evening. The resorption begins very soon after the roots of the teeth are complete; the building up is very quickly followed by the tearing down. The roots of the temporary teeth are completed between the fourth and sixth years. The sum and substance of the problem is to prevent the entrance of pyogenic organisms to the apical portions of the pericementum, and this means constant care.

*Dr. A. H. Porter.*—We talk here of chemistry as though it were the basis. We should look also to the physical condition of the teeth. Chemistry is but a subdivision of physics. I think we should look to the electrical conditions between our fillings and the teeth. We have heterogeneous substances present, and why not electric currents? Lord Kelvin says he has studied electricity for fifty years, and even now knows nothing about it. We are ignorant of the deeper meanings of this question of electricity.

I believe in mixing carbolic acid with zinc phosphate when using it in children's teeth.

*Dr. Register.*—The question is in the treatment of the temporary teeth to prevent local septicæmia. If we do that resorption can go on. I do not believe in using oxychloride or cotton dressings, for when exposed by resorption they are irritating. It is my practice to use salol.

*Dr. Guilford.*—Before reading my paper I felt like apologizing for its elementary character. I had no new views to present upon the subject, and chose it only because it is one seldom considered at dental meetings, but yet one of great importance to all practitioners.

I feared that what I might have to say would provoke no discussion, but have been pleased to find so many ready to express their views.

The council asked me to write a *short* paper, and so I felt that I had to be brief, and therefore could not touch upon some points or elaborate others. Nitrate of silver has certainly proven itself of great value in checking caries in the deciduous teeth, and should be employed more frequently than it is.

I should, perhaps, have mentioned that I use arsenious acid for the devitalization of deciduous pulps, just as I do for those of the permanent teeth, but I apply it in the minutest quantity and do not allow it to remain longer than from six to twelve hours. I have never seen any ill results from its use when used sparingly and carefully.

I was pleased with Dr. Burchard's explanation of why the roots of devitalized teeth were not resorbed in some cases and were in others. The idea was new to me, but I believe his reasoning to be correct.

Adjourned.

HENRY H. BURCHARD,
*Editor Academy of Stomatolgy.*

